# Mediterranean Fever Gene Analysis in The Azeri Turk Population with Familial Mediterranean Fever: Evidence for New Mutations Associated with Disease

**Published:** 2013-07-02

**Authors:** Leila Mohammadnejad, Safar Farajnia

**Affiliations:** 1 Biotechnology Research Center, Tabriz University of Medical Sciences, Tabriz, Iran; 2 Drug Applied Research Center, Tabriz University of Medical Sciences, Tabriz, Iran

**Keywords:** Familial Mediterranean Fever, *MEFV* Gene, Mutation, PCR, Sequence Analysis

## Abstract

**Objective:**

Familial Mediterranean fever (*FMF*) is an autosomal recessive disorder characterized by recurrent febrile attacks accompanied by serosal and synovial membrane inflammation. *FMF* is caused by mutations in the *MEFV* gene and are found usually among Mediterranean populations, Armenians, Turks, Arabs and Jews. The aim of this study was to determine the frequency of *MEFV* gene mutations among *FMF* patients in the Azeri Turk population in North-West of Iran.

**Materials and Methods:**

In this descriptive study, 130 *FMF* patients with Azeri Turk origin were screened for mutations in four exons (2, 3, 5 and10) of *MEFV* gene. Genomic DNA was extracted from whole blood and entered in ARMS-PCR and PCR-RFLP reactions. When cases were negative in ARMS-PCR and PCR-RFLP, the exons were amplified and subjected to direct sequencing.

**Results:**

Our results showed that the most common mutations in this study population was *M694V* (40.19%) followed by *E148Q* (17.64%), *V726A* (13.72%), *M680I* (12.74%) and *M694I* (2.94%) mutations. Four new mutations including *K618N*, K716M, S614F and G136E were identified in our study.

**Conclusion:**

The prevalence of five common mutations in our study was highly similar to previous studies analysing the Mediterranean basin populations. Investigation by sequencing also revealed four new variants in the study population. The main genotypephenotype correlation finding was the presence of *M694V* mutation in homozygote or compound heterozygote state in the patients with renal manifestations.

## Introduction

Familial Mediterranean fever (*FMF*), the most common hereditary periodic fever, is an autosomal recessive acute inflammatory disorder characterized by relapsing self-resolving febrile attacks and inflammation. It is accompanied by peritonitis, pleuritis, arthritis, skin rash and pain. The most severe complication of *FMF* is secondary amiloidosis commonly influencing the kidneys (11% of cases), and sometimes the adrenals, intestine, spleen, lung and testis (1-3). Moreover, clinical characteristics of the disease are different among patients from different ethnic groups (4). *FMF* mainly affects people from the Mediterranean basin countries especially Turks, Arabs, Armenians, and Sephardic Jews with a genetic prevalence of 6-8%. However, sporadic cases are also reported from other ethnicities (1, 2, 5). The carrier frequency for *MEFV* mutations in the populations that are more affected is very high, ranging from 37-39% in Armenians and Iraqi Jews and up to 20% in Turks, North African and Ashkenazi Jews, and Arabs. Despite high carrier prevalence in these populations, the frequency of *FMF* is less than anticipated, implying that the disease is either under diagnosed or that disease-related mutations have low penetrance (6). Until recently, the diagnosis of *FMF* was based on clinical signs, ethnicity, family history and response to colchicine. The identification of *FMF* causing gene (*MEFV*) has led to many studies analyzing the frequency of various mutations in different populations (7). The gene responsible for *FMF* (*MEFV*) is located on chromosome 16p13.3 and includes 10 exons. *MEFV* encodes a 781-aminoacid protein termed pyrin/marenostrin which probably assists the negative regulation of granulocytemediated inflammation. Nowadays, more than 200 sequence variants have been reported in the *MEFV* gene but not all are pathologic (1, 2). Most of these mutations are substitutions in exon 10. Five most commonly observed mutations i.e *M694V*, *M680I*, *M694I*, *V726A* and *E148Q* are responsible for a large percentage (about 65-95%) of observed mutations in different ethnic groups (1).

Iran is a country with different ethnic groups, including Persian (51%), Azeri Turk (24%), Kurd (7%), Arab (3%), and other smaller groups, such as Armenian. Although there are several *FMF* susceptible ethnic groups in Iran, the prevalence of *FMF* related mutations in the Iranian population has not been well defined. Only few *MEFV* gene mutational studies have been carried out about common FMF mutations in the Iranian population (5, 8-10). In this study the frequency of mutations in 4 exons of *MEFV* gene were investigated in clinically diagnosed *FMF* patients of Azeri Turk origin.

## Materials and Methods

### Patients

This descriptive study was carried out in the molecular biology lab in Tabriz biotechnology research center over a three year period of 2008-2010. The subjects include 130 (78 males, 52 females) Azeri Turk individuals living in the North West region of Iran. The subjects included 117 patients who fulfilled published diagnostic criteria for *FMF* and 13 of their asymptomatic first-degree relatives. The clinical inclusion and exclusion criteria were based on the standard Tell Hoshomer criteria for *FMF* diagnosis (2). A complete medical report and family history was collected for each individual and all of them provided informed consent before entering the study. This research was approved by the Ethical Committee of Tabriz University of Medical Sciences.

### DNA extraction and PCR analysis

DNA was extracted from peripheral blood leukocytes of the patients by standard methods (5, 11). According to previous studies, ARMSPCR (amplification refractory mutation system- PCR) and PCR-RFLP (PCR-restriction fragment length polymorphism) techniques were reliable methods to detect the point mutations (12, 13). Accordingly, we decided to use these techniques for detection of common *MEFV* mutations. Four common mutations in exon 10 (*Met694Val, Met680Ile, Val726Ala, Met694Ile*) were investigated by ARMS PCR and *E148Q* mutation in exon 2 was detected by PCR-RFLP using Ava1 restriction enzyme (Fermentas, Lithuania). The primers were designed by Oligo software version 5.0 and the expected product sizes are shown in table 1. Polymerase chain reaction (PCR) was performed in 25 microliter reaction volumes containing 100 ng genomic DNA, 25 pmols primers (MWG, Germany), 0.2 mM dNTPs (Fermentas, Lithuania), 2.5 mL reaction buffer (Fermentas, Lithuania) and 1U Taq DNA polymerase (Fermentas, Lithuania). Cycling conditions were 94˚C, four minutes, followed by 30 cycles of denaturation at 94˚C, one minute, annealing (at 58˚C for *M694V* and *V726A*, 66˚C for *M680I*, 64˚C for *M694I* and 63˚C for*E148Q*), 30 seconds, extension at 72˚C for 30 seconds and a final extension of 72˚C for 5 minutes. PCR reactions were carried out in a thermo cycler (Eppendorf, Germany). The proper positive and negative controls were used for each reaction. PCR products and restriction enzyme-digested fragments were separated by electrophoresis on a 2% agarose gel (Sigma Aldrich, Germany). Ethidium bromide staining of the agarose gel was used to detect the amplified fragments. The results of PCR-RFLP and ARMS-PCR were checked by sequencing of randomly selected samples.

**Table 1:** The sequence of oligonucleotides used in ARMS-PCR and PCR-RFLP methods and expected product sizes


Primer name	Sequence	Expected product size

**M694V common**	5'-TATCATTGTTCTGGGCTC-3'	183 bp
**Mutant**	5'-TGGTACTCATTTTCCTTCAC-3'	
**Normal**	5'-TGGTACTCATTTTCCTTCAT-3'	
**M694I common**	5'-TATCATTGTTCTGGGCTC-3'	183 bp
**Mutant**	5'-CTGGTACTCATTTTCCTTT-3'	
**Normal**	5'-CTGGTACTCATTTTCCTTC-3'	
**M680I common**	5'- GGAAACAAGTGGGAGAGGCTGC-3'	197 bp
**Mutant**	5'- GTAGCCATTCTCTAGCGACAGTGCC -3'	
**Normal**	5'- GTAGCCATTCTCTAGCGACAGTGCG -3'	
**V726A common**	5'- TTGGAGACAAGACAGCATGGATCC-3'	230 bp
**Mutant**	5'- GTCACATTGTAAAAGGAGATGCTTGCTG-3'	
**Normal**	5'-CTGTCACATTGTAAAAGGAGATGCTTGCTA-3'	
**E148Q forward**	5´- ATATTCCACACAAGAAAACGGC-3´	247 bp
**E148Q reverse**	5´- GAGGCTTGCCCTGCGCG-3´	


### Direct Sequencing

Direct sequencing was used for samples with negative results in ARMS-PCR and PCR-RFLP. Entire exons (2, 3, 5, 10) were amplified and subjected to sequencing by dideoxy method using sequencing primers (Table 2). The sequencing results were compared with the *MEFV* reference coding sequence available at NCBI with GenBank accession number AF111163.

**Table 2:** The sequence of primers used in direct sequencingmethod


**FMF-E2-F**	5´- TTGCATCTGGTTGTCCTTCC - 3´
**FMF-E2-R**	5´- CCGATATAAAGTAGGAAAGAACAC- 3´
**FMF-E3-F**	5´-’TCCACTGCATGTCCCCAGG-3´
**FMF-E3-R**	5´- CAAGTGCCTGGCAGAGAAGAGC-3´
**FMF-E5-F**	5´- CATACTGATAGGCACAGGGGACC -3´
**FMF-E5-R**	5´- TCCACGTCCACCCACAGCAC -3´
**FMF-E10-F**	5´-CCCATGGACCCCTACCTAGG- 3´
**FMF-E10-R**	5´-AAGAGAGATGCAGTGTTGGGC-3´


## Results

### Clinical criteria

According to the recorded demographic data, all of the patients were from Azeri Turk origin. The age range of subjects was between 4 months to 51years (mean of 22 ± 14.4 years) and the mean age of onset was 5.2 ± 3.9 years. Analysis of clinical symptoms according to the Tel-Hashomer criteria (2) showed a classic pattern where fever (84.03%) and peritonitis (78.15%) were the most common clinical symptoms. Clinical criteria is summarized in table 3.

**Table 3:** Frequency of FMF symptoms in our cohort study


Clinical symptom	Fever	Peritonitis	Pleuritis	Arthritis	Renal manifestations	Erysipelas-like erhythema

**No (%)**	100 (84.03)	93 (78.15)	50 (42.01)	58 (48.73)	19 (15.96)	7 (5.88)


### MEFV genotyping

**Fig 1:**
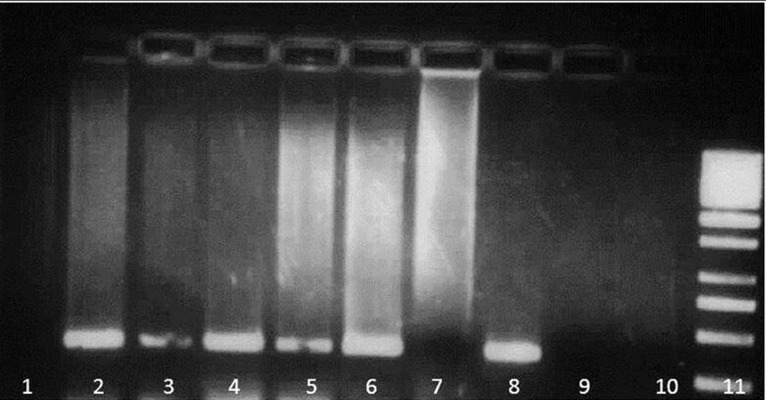
ARMS-PCR result for M694V mutation. Detection of four common MEFV gene mutations by ARMS-PCR. For each mutation, the ARMS assay consists of two PCR reactions specific for the normal and mutant alleles. Lane1, 3, 5 and 7 are reactions for mutant alleles and lanes 2, 4, 6 and 8 are reactions for normal alleles. Lane 9 and 10 are for no DNA controls and lane 11 is the DNA ladder.

**Fig 2:**
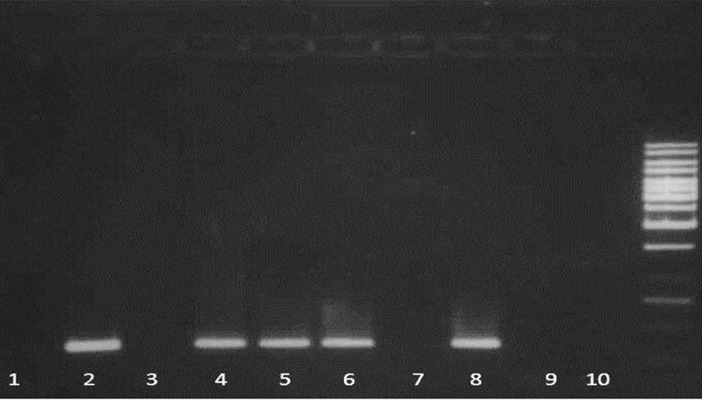
ARMS-PCR result for M680I mutation. Detection of four common MEFV gene mutations by ARMSPCR. For each mutation, the ARMS assay consists of two PCR reactions specific for the normal and mutant alleles. Lane1, 3, 5 and 7 are reactions for mutant alleles and lanes 2, 4, 6 and 8 are reactions for normal alleles. Lane 9 and 10 are for no DNA controls and lane 11 is the DNA ladder.

**Fig 3:**
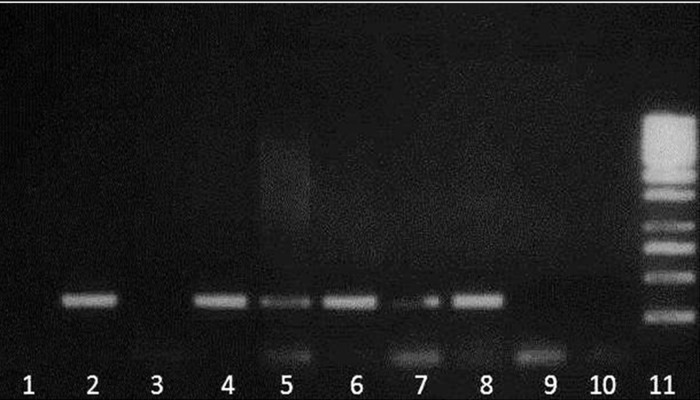
ARMS-PCR result for V726A mutation. Detection of four common MEFV gene mutations by ARMS-PCR. For each mutation, the ARMS assay consists of two PCR reactions specific for the normal and mutant alleles. Lane1, 3, 5 and 7 are reactions for mutant alleles and lanes 2, 4, 6 and 8 are reactions for normal alleles. Lane 9 and 10 are for no DNA controls and lane 11 is the DNA ladder.

**Fig 4:**
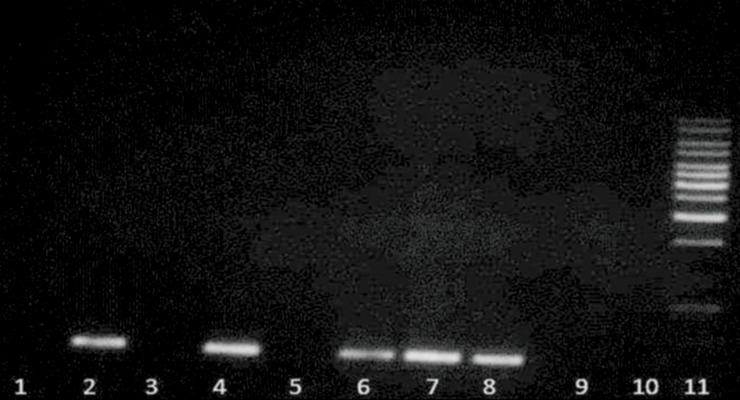
ARMS-PCR result for M694I mutation. Detection of four common MEFV gene mutations by ARMS-PCR. For each mutation, the ARMS assay consists of two PCR reactions specific for the normal and mutant alleles. Lane1, 3, 5 and 7 are reactions for mutant alleles and lanes 2, 4, 6 and 8 are reactions for normal alleles. Lane 9 and 10 are for no DNA controls and lane 11 is the DNA ladder.

**Fig 5:**
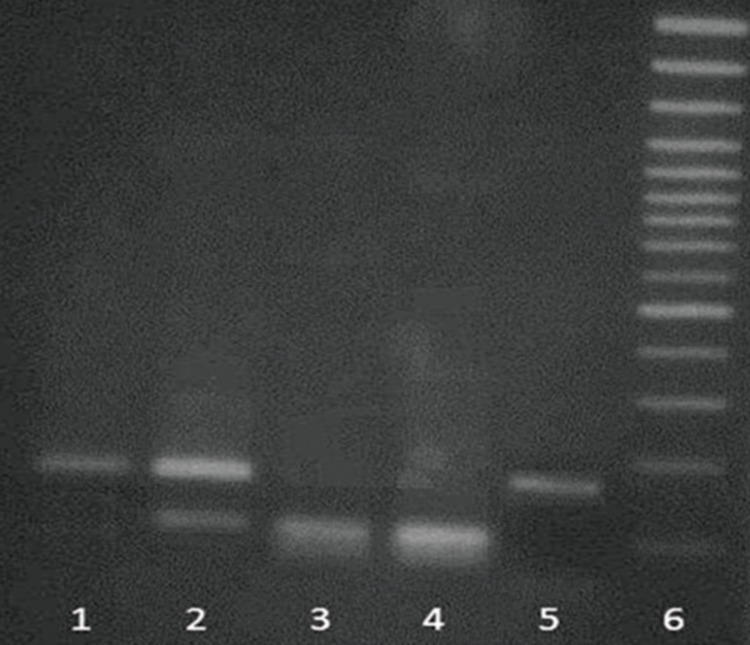
PCR-RFLP analysis of E148-Q mutation in FMF patients. Lane 1. undigested PCR product; lane 2, mutant heterozygote; lanes 3 and 4, mutant homozygote, lane 5, normal and lane 6. size marker.

Molecular diagnosis of mutations was carried out by ARMS-PCR, PCR-RFLP and sequencing methods. A total of twenty one different genotypes were identified between 78 *FMF* patients. No *MEFV* mutations were found in 52 cases. Among 117 cases with *FMF* diagnosis and 13 asymptomatic relatives, 28 (21.53%) patients had homozygote mutations, 26 (19.95%) cases were found to have one heterozygote mutation and the remaining 24 (18.46%) were compound heterozygotes (Table 4). Four healthy relatives of patients were found to carry one heterozygote *MEFV* mutation (*M694V* and *M680I*), two of them were parents of an affected child with *M694V*/ *M680I* compound heterozygote genotype. Two remaining relatives were parents of two patients with single heterozygote *M694V* and *M680I* mutations. Figures 1-5 show mutation analysis for 5 common *MEFV* gene mutations by ARMS-PCR and PCR-RFLP.

**Table 4:** MEFV genotypes in 130 FMF patients from North-West of Iran


Mutation	Genotype	Number (%)

**Heterozygote**	M694V/-	6 (4.61)
	E148Q/-	8(6.15)
	V726A/-	3 (2.3)
	M694I/-	1 (0.76)
	R761H/-	2 (1.53)
	A744S/-	2 (1.53)
**Compound heterozygote**	new variant /-	4 (3.07)
	M694V/V726A	6 (4.61)
	M694V/M680I	6 (4.61)
	M694V/E148Q	4 (3.07)
	M680I/V726A	2 (1.53)
	V726A/E148Q	2 (1.53)
	M694V/M694I	2 (1.53)
	G632A / new variant	2 (1.53)

**Homozygote**	M694V	17 (13.07)
	M680I	5 (3.84)
	E148Q	4 (3.07)
	V726A	1 (0.76)
	New variant	1 (0.76)
**Total patients with mutation**		78 (60)

*M694V* accounted for the majority of *FMF* mutations with a frequency of 40.19% followed by*E148Q* (17.64%), *V726A* (13.72%), *M680I* (12.74%) and *M694I* (2.94%). Other/ novel mutations detected by complete exon sequencing were found in 11(8.42%) cases as shown in table 4. The results also indicated that five common missense mutations namely M64V, *M680I*, *M694I*, *V726A* and *E148Q* accounted for 87.25% of detected *MEFV* mutations.

### Sequencing analysis

In the course of screening via direct sequencing, seven different mutations were found in 11 patients with negative results in ARMS- and RFLP- PCR. Three of these mutations i.e. *A744S*, *G632A* and *R761H* have been reported in previous studies, whereas four mutations were novel to this study. Nucleotide change 1853G>C causing lysine-to-asparagine substitution in codon 618 (*K618N*) was detected in homozygote and heterozygote form in one and three symptomatic *FMF* cases respectively. Also this new variant was found in a compound heterozygote state with *G632A*, a previously reported mutation, in two patients. Patient affected by *K618N* homozygote mutation have a severe form of disease whereas other new mutations was found in the symptomatic patients with mild to moderate state. All other novel variants were found on only one *MEFV* allele in the symptomatic patients as summarized in table 5.

**Table 5:** The list of novel variants of MEFV gene found in this study


Nucleotide change	Amino acid change	State	Number of affected patients	Number of exon

**2147A>T**	K716 M	Heterozygote	1	10
**1853G>C**	K618N	Heterozygote	3	10
**1853G>C**	K618N	Homozygote	1	10
**1841C>T**	S614F	Heterozygote	1	10
**407G>A**	G136E	Heterozygote	1	2


### Genotype-phenotype correlation

Among 13 cases with renal manifestation, six patients were homozygote for the *M694V* mutation and 2 cases were *M680I* homozygotes. The remaining 5 affected people were compound heterozygotes with the following genotypes: *M694V*/*M680I* in 2 cases, *M694V*/*M694I*, *M694V*/*V726A* and *G632A*/*K618N* in one patient. The results also indicated that homozygote *M694V* mutation is associated with the severe form of the disease (p<0.05). However, there was no significant association between other clinical criteria and the specific mutations (p>0.05).

## Discussion

The allele frequency of *MEFV* mutations varies among ethnic groups. The Iranian Azeri Turk ethnicity is considered to be a susceptible population to *FMF*, but there are scant reports on the prevalence of *MEFV* mutations in this population (1, 2, 9, 14). In the present study we have analyzed the frequency of *MEFV* mutations in Azeri Turk population living in North West of Iran. This is the first study that was undertaken by sequencing method for mutation analysis of four *MEFV* exons in this ethnic group.

The results of our study indicated that *M694V* variant was the most common mutation (40.19%) in this cohort study, followed by *E148Q* (17.64%), *V726A* (13.72%), *M680I* (12.74%) and *M694I* (2.94%). These results are consistent with previous studies in Azeri Turk population by PCR and Strip assay methods (8- 10). However in a study on 36 *FMF* patients in the central part of Iran (Tehran city), the most common mutations were *M680I*, *M694V*, *V726A*, *E148Q* and *M694I* with the frequency of 23.6, 22.6, 15.3, 6.9 and 2.8%, respectively (10). The different frequency distribution of mutations may be related to the small sample size or genetic heterogeneity. Also the mutation frequency in our study group was in agreement with studies in Turks (3, 15-18), Armenians (2, 19, 20) and Jews (3, 21-23). *M694V*, *V726A*, *E148Q* and *M680I*, which were the four most common mutations in our cohort study, were also the most frequent reported mutations in Mediterranean populations (2, 3). The genotype frequency in our study and their comparison to other studies are summarized in table 6.

It has been shown that some rare *MEFV* mutations tend to be over represented in particular ethnic groups, but have been sporadically seen in other populations. For instance, *R761H* is more prevalent in Armenians and Turks, *K695R* in Jews, *A744S* in Arabs and *F479L* in Armenians (1). Screening by sequencing found seven rare mutations in our study population, where three of them (*R761H*, *A744S*, *G632A*) had previously been reported in *FMF* subjects in other studies and four mutations (*K716 M, K618N, S614F, G136E*) were novel. K618M mutation was detected in homozygote once and compound heterozygote state in three symptomatic *FMF* patients. The other three mutations were found in heterozygote form in FMF affected patients in the absence of any other mutations. All of these new variants were found in patients with clinical symptoms characteristic of FMF. Two of these novel changes were discovered in compound heterozygote form with *G632A*, a mutation known to be associated with *FMF*. Also no other mutations were identified in the complete exon sequencing of *MEFV* in patients affected with one novel heterozygote mutation. Consequently, these changes may be considered as *FMF* causes mutation and recommended to study in future studies. Also, the relevance of these novel mutations to *FMF* should be confirmed by studying in a large patients group and control subjects in different ethnic groups.

Different studies reported a significant association between *M694V* mutation and the severity of disease (3, 27, 28). It has been shown that patients with homozygote *M694V* mutation have an earlier onset and higher frequency of arthritis compared to the other genotypes. Also the prevalence of renal amyloidosis is higher in *M694V* homozygous patients than in patients with other *MEFV* genotypes (29-31). In this study we found an association between *M694V* mutation and severity of the disease and renal manifestation. But, we did not find any association between specific mutation or genotypes and other clinical features, such as, age of onset, attack frequency etc.

The presence of only one *MEFV* mutation in clinically diagnosed *FMF* patients have always been a subject of concern. At first, some researchers supposed the presence of mutations in the second *MEFV* allele, but a number of studies have not detected any other mutation in the complete gene sequencing (18, 32). It has been suggested that modifying genes such as major histocompatibility complex (*MHC*) class-I-chainrelated gene A (*MICA*) and serum amyloid A (SAA) could be a possible reason for such observations. In some cases, *FMF* has been reported as a dominant state with low penetrance (6, 33). Our results are consistent with the hypothesis about the clinical implications of some *FMF* mutations in heterozygous forms. The frequency of heterozygote subjects in our study was 19.95%. So it seems that, the presence of a given mutation is enough to cause *FMF* clinical symptoms in some patients.

**Table 6:** The MEFV mutation frequency in our cohort study in comparison to other study populations


Descent/ references	M694V	E148Q	V726A	M680I	M694I	Other/new mutations

**Turkish (3, 15-18)**	45 (41-73)	3.5 (1-13)	11 (2-14)	13 (6-31)	7 (0-14)	1 (0-3)
**Jewish (3, 21-23)**	65 (56-100)	5 (4-10)	3 (0-12)	1 (0-8)	0 (0-1)	6 (2-10)
**Armenian (3, 19, 20)**	37 (21-52)	3 (1-11)	19 (11-26)	20 (5-27)	2 (0-10)	2 (1-5)
**Arabs (3, 24-26)**	20 (9-23)	6 (0-11)	14 (0-29)	7 (0-21)	12 (0-42)	3 (0-7)
**Iranian (8-10) **	39 (22-54)	12 (6-16)	16 (15-17)	17 (12-23)	2 (2-3)	19 (10-28)
**Our study**	40.19	17.64	13.72	12.74	2.94	8.42


## Conclusion

This study analyzed the spectrum of *MEFV* mutations among FMF patients of Azeri Turk origin in the North West region of Iran. A genotype-phenotype correlation showed an association between the *M694V* mutation and the severe form of the disease and renal manifestation. Also the results of our study revealed presence of novel mutations in Iranian Azeri Turk population.
